# The V2 receptor antagonist tolvaptan raises cytosolic calcium and prevents AQP2 trafficking and function: an *in vitro* and *in vivo* assessment

**DOI:** 10.1111/jcmm.13098

**Published:** 2017-03-21

**Authors:** Grazia Tamma, Annarita Di Mise, Marianna Ranieri, Ari Geller, Roberto Tamma, Alberta Zallone, Giovanna Valenti

**Affiliations:** ^1^ Department of Biosciences Biotechnologies and Biopharmaceutics University of Bari Aldo Moro Bari Italy; ^2^ St Francis Hospital Hartford CT USA; ^3^ Department of Basic Medical Science Neuroscience and Sense Organs University of Bari Aldo Moro Bari Italy

**Keywords:** tolvaptan, aquaporin‐2, SIAD, vasopressin, hyponatraemia, PKD

## Abstract

Tolvaptan, a selective vasopressin V2 receptor antagonist, is a new generation diuretic. Its clinical efficacy is in principle due to impaired vasopressin‐regulated water reabsorption *via* aquaporin‐2 (AQP2). Nevertheless, no direct *in vitro* evidence that tolvaptan prevents AQP2‐mediated water transport, nor that this pathway is targeted *in vivo* in patients with syndrome of inappropriate antidiuresis (SIAD) has been provided. The effects of tolvaptan on the vasopressin–cAMP/PKA signalling cascade were investigated in MDCK cells expressing endogenous V2R and in mouse kidney. In MDCK, tolvaptan prevented dDAVP‐induced increase in ser256‐AQP2 and osmotic water permeability. A similar effect on ser256‐AQP2 was found in V1aR −/− mice, thus confirming the V2R selectively. Of note, calcium calibration in MDCK showed that tolvaptan *per se* caused calcium mobilization from the endoplasmic reticulum resulting in a significant increase in basal intracellular calcium. This effect was only observed in cells expressing the V2R, indicating that it requires the tolvaptan–V2R interaction. Consistent with this finding, tolvaptan partially reduced the increase in ser256‐AQP2 and the water permeability in response to forskolin, a direct activator of adenylyl cyclase (AC), suggesting that the increase in intracellular calcium is associated with an inhibition of the calcium‐inhibitable AC type VI. Furthermore, tolvaptan treatment reduced AQP2 excretion in two SIAD patients and normalized plasma sodium concentration. These data represent the first detailed demonstration of the central role of AQP2 blockade in the aquaretic effect of tolvaptan and underscore a novel effect in raising intracellular calcium that can be of significant clinical relevance.

## Introduction

Tolvaptan is a selective vasopressin V2 receptor (V2R) antagonist that was approved by the Food and Drug Administration (FDA) in 2009 for the treatment of euvolemic and hypervolemic hyponatraemia (serum sodium < 125 mEq/l), associated with heart failure, cirrhosis and the SIAD 1, 2, 3. Recent studies have also suggested tolvaptan as a promising therapeutic tool in autosomal dominant polycystic kidney disease (ADPKD) 4. Compared with other vaptans, tolvaptan can bind V2R with 1,8 higher affinity than the native hormone vasopressin and it is 29‐fold more selective for V2R with respect to V1A receptor 5. In the kidney, vasopressin binds its cognate V2R receptor, activating the cAMP signal transduction cascade, leading to the translocation of the AQP2‐bearing vesicles to the apical plasma membrane, resulting in water reabsorption 6, 7. This vesicle transport is associated with an increase in the phosphorylation of AQP2 at serine 256 (pS256), secondary to cAMP‐dependent kinase (PKA) activation 8, 9. By blocking V2R, tolvaptan causes a relevant aquaresis, a consequent decrease in urinary osmolality and a rapid correction of serum natraemia. In rats, prolonged subcutaneous infusion of tolvaptan significantly decreased the abundance of β and γ subunits of ENaC, of AQP3 and of AQP2 which was less phosphorylated at S256, S264 and S269 compared with vehicle infused rats 10.

Tolvaptan has been shown to significantly reduce cAMP levels in an orthologous rat model of human cystic disease 11. Conversely, a V2R agonist increased renal cAMP and cystogenesis. Data from completed clinical trials revealed that tolvaptan reduced kidney weight, enlargement of the cysts and PKD progression 4. However, the molecular details have not been fully elucidated.

The present study was undertaken to investigate the molecular basis of the tolvaptan effect in collecting duct principal cells. To this end, MDCK cells expressing endogenous V2R receptor and stably transfected with AQP2 were used.

The major novel finding is the observation that tolvaptan *per se* causes calcium mobilization from the ER resulting in a significant increase in basal intracellular calcium, an effect which can be of significant clinical relevance. Furthermore, we provide here the first demonstration of the central role of AQP2 blockade in the aquaretic effect of tolvaptan both *in vitro* and *in vivo* in two SIAD patients.

## Materials and methods

### Chemicals and reagents

All chemicals were purchased from Sigma (Sigma‐Aldrich, Milan, Italy). Fura‐2‐AM and calcein‐AM were bought from Molecular Probes (Life Technologies, Monza, Italy). Tolvaptan was kindly gifted from Otsuka (Otsuka Pharmaceutical Co., Ltd, Tokyo Japan). Forskolin was purchased from Fermentek (Jerusalem, Israel). Media for cell culture were from PAA (GE Healthcare Life Sciences, Piscataway, NJ, USA). Phosphatases activity assay kit was purchased from Millipore (Billerica, MA).

### Antibodies

Total AQP2 was detected with antibodies (Pre‐C‐tail Ab) against the 20‐amino acid residue segment just N‐terminal from the polyphosphorylated region of rat AQP2 (CLKGLEPDTDWEEREVRRRQ) 12, 13. Alternatively, AQP2 was detected with a specific antibody (C‐tail Ab) raised against a synthetic peptide corresponding to the last 15 C‐terminal amino acids of human AQP2 14. AQP2‐pS256 antibodies were kindly gifted by Peter Deen and described by Trimpert *et al*. 15. Monoclonal antibody against glyceraldehyde‐3‐phosphate dehydrogenase (GAPDH, clone 6C5) was purchased from Millipore (Millipore Corporation). Secondary goat anti‐rabbit and antimouse‐Alexa488‐conjugated antibodies were from Molecular Probes (Eugene, OR, USA).

### Cell culture and treatments

MDCK‐hAQP2 type I cells, stably expressing human AQP2, were grown as described 16. In brief, cells were grown in Dulbecco's modified Eagle's medium (DMEM) high glucose, supplemented with 5% (v/v) foetal bovine serum, 1% (v/v) L‐glutamine, 1% (v/v) non‐essential amino acids and 0.5% ciprofloxacin, at 37°C in 5% CO_2_. After overnight treatment with indomethacin (5 × 10^−5^ M), cells were left under basal condition or stimulated with 100 nM dDAVP for 45 min., 10 nM tolvaptan for 45 min., 10 μM forskolin (FK) for 45 min. or 10 nM SR49059, a selective arginine vasopressin V1a receptor antagonist, for 15 min. As reported 16, addition of the prostaglandin synthesis inhibitor indomethacin was needed to reduce basal cAMP and AQP2‐pS256 levels 16, 17 and was present in all treatments.

### Cell preparations

MDCK‐hAQP2 cells were seeded onto 60‐mm dishes and left under basal conditions or stimulated. Then, cells were homogenized in cell fractionation buffer (20 mM NaCl, 130 mM KCl, 1 mM MgCl2, 10 mM HEPES, pH 7.5) in the presence of proteases (1 mM PMSF, 2 mg/ml leupeptin and 2 mg/ml pepstatin A) and phosphatases (10 mM NaF and 1 mM sodium orthovanadate) inhibitors. The obtained homogenates were sonicated at 80% amplitude for 10 sec. Cellular debris was removed by centrifugation at 12,000 × *g* for 10 min. at 4°C. The supernatants were collected and used for immunoblotting studies.

### *Ex vivo* preparation

Kidney slices from mouse papilla were prepared as described 18. Kidneys were quickly removed, and sections of approximately 500 μm were made. Sectioned kidney papillae were equilibrated for 10 min. in a buffer containing 118 mM NaCl, 16 mM HEPES, 17 mM Na‐HEPES, 14 mM glucose, 3.2 mM KCl, 2.5 mM CaCl_2_, 1.8 mM MgSO_4_ and 1.8 mM KH_2_PO_4_ (pH 7.4). Subsequently, kidney slices were left in the same buffer at 37°C or incubated with 100 nM dDAVP for 45 min., 100 nM dDAVP and 10 nM tolvaptan for 45 min., 10 μM FK for 45 min., or 10 μM FK and 10 nM tolvaptan for 45 min. The treated sections were then homogenized with a mini‐potter on ice‐cold buffer containing 220 mM mannitol, 70 mM sucrose, 5 mM EGTA, 1 mM EDTA and 20 mM Tris–HCl, pH 7.4, and protease and phosphatase inhibitors. Suspensions were sonicated and centrifuged at 12,000 × *g* for 10 min. at 4°C. The supernatants were assayed for Western blotting.

### Gel electrophoresis and immunoblotting

Proteins were separated on 13% Bis‐Tris acrylamide gels under reducing conditions. Protein bands were electrophoretically transferred onto Immobilon‐P membranes (Millipore Corporate Headquarters, Billerica, MA, USA) for Western blot analysis, blocked in TBS–Tween‐20 containing 3% BSA and incubated with primary antibodies O/N. Anti‐AQP2 (Pre‐C‐tail Ab) and anti‐AQP2‐pS256 were used at 1:1000 dilution; anti‐GAPDH was used at 1:5000 dilution. Immunoreactive bands were detected with secondary goat anti‐rabbit or goat antimouse horseradish peroxidase‐coupled antibodies obtained from Santa Cruz Biotechnologies (Tebu‐Bio, Milan, Italy). Membranes were developed with SuperSignal West Pico Chemiluminescent Substrate (Pierce, Rockford, IL, USA) with Chemidoc System (Bio‐Rad Laboratories, Milan, Italy). Representative figures are shown. Densitometry analysis was performed with Scion Image. Data are summarized in histograms with GraphPad Prism (Graphpad Software Inc. La Jolla, CA, USA).

### Immunofluorescence

Immunofluorescence localization of AQP2 in MDCK‐hAQP2 was performed as previously described 19. Briefly, MDCK‐hAQP2 cells were grown on cell culture PET inserts, treated as described above and fixed for 20 min. with 4% paraformaldehyde in PBS. Samples were permeabilized with 0.1% Triton X‐100 in PBS for 5 min., blocked with 1% BSA–PBS for 45 min. and incubated with a 1:1000 dilution of AQP2 antibodies for 2 hrs. After washing three times with 1% BSA–PBS, samples were incubated with 1:1000 diluted goat anti‐rabbit antibodies coupled to Alexa‐488 in 1% BSA–PBS for 1 hr. Next, cells were rinsed three times with PBS and mounted on glass slides with Mowiol. Images were obtained with a confocal laser scanning fluorescence microscope Leica TCS SP2 (Leica Microsystems, Heerbrugg, Switzerland).

### Video imaging intracellular calcium measurements

For intracellular Ca^2+^ measurements, cells were grown on Ø40‐mm glass coverslips. Cells were loaded with 7 μM fura‐2‐AM for 15 min. at 37°C, 5% CO_2_ in DMEM. During experiment, cells were perfused with Ringer's solution containing 137 mM NaCl, 5.4 mM KCl, 0.5 mM MgCl_2_, 4.2 mM NaHCO_3_, 3 mM Na_2_HPO_4_, 0.4 mM KH_2_PO_4_, 10 mM HEPES sulphonic acid, 10 mM glucose and 1.3 mM CaCl_2_, pH 7.4. In fluorescence measurements, coverslips with dye‐loaded cells were mounted in a perfusion chamber (FCS2 Closed Chamber System; BIOPTECHS, Butler, PA, USA) and measurements were taken with an inverted microscope (Nikon Eclipse TE2000‐S microscope; Nikon Inc. Melville, NY, USA) equipped for single‐cell fluorescence measurements and imaging analysis. The sample was illuminated through a 40× oil immersion objective (NA = 1.30). The fura‐2‐AM‐loaded sample was excited at 340 and 380 nm. Emitted fluorescence was passed through a dichroic mirror, filtered at 510 nm (Omega Optical, Brattleboro, VT, USA) and captured by a cooled CCD camera (CoolSNAP HQ, Photometrics Inc. Huntington Beach, CA, USA). Fluorescence measurements were taken with Metafluor software (Molecular Devices, MDS Analytical Technologies, Toronto, Canada). Intracellular calcium level was calculated as described by Grynkiewicz 20. Briefly, calcium concentration was determined from the emission fluorescence ratio of the two excitation wavelengths according to the formula (Ca^2+^)_i_ = K_d_*Q(*R* − *R*_min_)/(R_max_ − *R*), where K_d_ (224 nM) indicates the dissociation constant of fura‐2‐AM for (Ca^2+^)_i_ and Q indicates the ratio of the fluorescence intensities (F) at the minimum and the maximum calcium concentration at 380 nm. Each sample was calibrated by the addition of 5 μM ionomycin in the presence of 1 mM EGTA (*R*_min_) followed by 5 μM ionomycin in 5 mM CaCl_2_ (*R*_max_).

### Fluorescence resonance energy transfer measurements

To evaluate intracellular cAMP levels and calcium content in the endoplasmic reticulum (ER), fluorescence resonance energy transfer (FRET) experiments were performed. Briefly, MDCK‐hAQP2 cells were seeded onto 20‐mm glass coverslips at 37°C, 5% CO_2_ and transiently transfected with 0.4 μg of DNA/cm^2^, with Lipofectamine (1 μg/μl) according to the protocol provided by the manufacturer (Life Technologies). For cAMP evaluation, cells were transfected with a plasmid encoding the H96 probe containing cAMP‐binding sequence of Epac1 between cyan fluorescent protein (CFP) and cp^173^Venus‐Venus (gift from Dr. K. Jalink) 21. Alternatively, cAMP changes were measured in the sub‐plasma‐membrane compartment with a membrane‐targeted version of H30 (mpH30) 22.

For ER calcium levels measurements, cells were transiently transfected with a plasmid encoding the D1ER Cameleon (gift from Prof. Roger Tsien) 23. Experiments were performed 48 hrs after transfection. Visualization of ECFP‐ and/or EYFP‐expressing cells and detection of FRET was performed on an inverted microscope (Nikon Eclipse TE2000‐S) equipped with a monochromator controlled by Metamorph/Metafluor software. ECFP was excited at 433 nm and EYFP at 512 nm. All images were aligned and corrected for background in the emission windows for FRET (535/30 nm), ECFP (475/30 nm) and EYFP (535/26 nm). Each image was further corrected for ECFP crosstalk and EYFP cross‐excitation as shown by Rodighiero *et al*. 24. Thus, netFRET = IFRETbg − ICFPbg × k1 − IYFPbg (K2‐aK1)]/(1‐K1d), where IFRETbg, ICFPbg and IYFPbg are the background‐corrected pixel grey values measured in the FRET, ECFP and EYFP windows, respectively; K1, K2, a and d are calculated to evaluate the crosstalk between donor and acceptor. The integrated fluorescence density values of the images from 10 regions of interest in each cell were analysed with Metamorph and Microsoft Excel software.

### Water permeability assay

Osmotic water permeability was measured by video imaging experiments. MDCK‐hAQP2 cells were grown on Ø40‐mm glass coverslips and loaded with 20 μM membrane‐permeable calcein‐AM for 45 min. at 37°C, 5% CO_2_ in DMEM. Cells were left under basal condition or stimulated with 100 nM dDAVP for 45 min. or with 100 nM dDAVP and 10 nM tolvaptan for 45 min. or with 100 nM dDAVP or with 10 nM tolvaptan for 45 min. or with 10 μM FK for 45 min. or with 10 μM FK in combination with 10 nM tolvaptan for 45 min. The coverslips with dye‐loaded cells were mounted in a perfusion chamber (FCS2 Closed Chamber System, BIOPTECHS), and measurements were taken with an inverted microscope (Nikon Eclipse TE2000‐S microscope) equipped for single‐cell fluorescence measurements and imaging analysis. The sample was illuminated through a 40× oil immersion objective (NA = 1.30). The calcein‐AM‐loaded sample was excited at 490 nm. Emitted fluorescence was passed through a dichroic mirror, filtered at 514 nm (Omega Optical, Brattleboro, VT, USA) and captured by a cooled ECCD camera (CoolSNAP HQ, Photometrics). Fluorescence measurements, following iso‐ (137 mM NaCl, 5.4 mM KCl, 0.5 mM MgCl_2_, 1.3 mM CaCl_2_, 4.2 mM NaHCO_3_, 3 mM Na_2_HPO_4_, 0.4 mM KH_2_PO_4_, 10 mM HEPES sulphonic acid, 10 mM glucose) or hyperosmotic (isosmotic solution added with 100 mM mannitol) solutions, taken with Metafluor software (Molecular Devices, MDS Analytical Technologies, Toronto, Canada). The time course of cell shrinkage was measured as time constant (*K*_*i*_, s^−1^), a parameter directly correlated with membrane water permeability.

### PP activities assay

The protocol used a PP1β activity assay kit as described 25. Cells were treated as mentioned above and lysed according to the protocol provided by the reagent manufacturer (Millipore Corporation, Billerica, USA). 300 μg proteins from cell lysate, determined with Qubit (Life Technologies, Monza, Italy), were incubated with 25 μl protein‐A agarose and 4 μl anti‐PP1β antibodies. After 2 hrs of incubation, immunocomplexes were washed three times with ice‐cold Tris‐buffered saline and one time with Ser/Thr phosphopeptide buffer. After the last wash, 60 μl diluted phosphopeptide (750 μM) and 20 μl phosphopeptide buffer were added and incubated for 10 min. at 30°C in a shaking incubator; 25 μl supernatant was placed in a 96‐well plate, and a malachite green detection assay was used to determine free phosphates. A calibration curve was generated to establish the level of phosphatase activity, which is reported in picomoles of phosphate released per 25 μl supernatant.

### Human subjects

Two patients were recruited in January 2013. Both patients were diagnosed with SIAD. Patient 1 had initial sodium of 129 mM and patient 2 had initial sodium of 115 mM. Approval for the study was obtained through the Institutional Review Board at Saint Francis Hospital and Medical Center in Hartford, CT. The inclusion criteria were adult patients (>18 years of age) with the diagnosis of hyponatraemia requiring treatment with tolvaptan. Exclusion criteria were patients less than 18 years of age. Hyponatraemia was defined as a serum sodium of less than 135 mM. Informed consent was obtained. Urine samples were collected before, 12 and 24 hrs after tolvaptan was administered.

### Urinary AQP2 measurements by ELISA (enzyme–linked immunosorbent assay)

Urinary AQP2 excretion was measured in urine samples by ELISA as previously described 26. Briefly, urine samples were spun at 600 × g for 10 min. at 4°C to remove cellular debris in the presence of the following protease inhibitors: 2 mM phenylmethylsulfonyl fluoride, 1 mg/ml leupeptin, 1 mg/ml pepstatin. 5 μl of urine sample was diluted to 50 μl in PBS containing 0.01% sodium dodecyl sulfate (SDS), placed in a MaxiSorp 96‐well microplate and incubated O/N at 4°C. In parallel wells, increasing concentrations (50, 100, 200, 300, 400, 500 and 1000 pg/50 μl) of a synthetic peptide reproducing the last 15 amino acids of the C‐terminal region of human AQP2 were incubated as internal standard. Each sample was analysed in triplicate. Wells were washed with washing buffer (PBS–0.1% Tween‐20) and incubated with a blocking solution of PBS containing 3% BSA at room temperature for 1 hr. 10 μg of affinity‐purified anti‐AQP2 antibodies was diluted in blocking solution (final antibody dilution 1:1000), and 50 μl of the solution was added to each well and incubated for 3 hrs at 37°C. Wells were then washed with washing buffer, and anti‐rabbit IgG conjugated with horseradish peroxidase was added to each well and incubated for 1 hr at 37°C. After five washings with washing buffer, 50 μl of the substrate solution [2,29‐azino‐bis(3‐ethylbenzothiazoline‐6‐sulphonic acid)] was added to each well and incubated for 30 min. in the dark. Absorbance was measured with a microplate reader (Model 550; Bio‐Rad) at 405 nm.

### Statistical analysis

One‐way anova followed by a Newman–Keuls multiple comparison test was used for the statistical analysis. All values are expressed as means ± S.E.Ms. A difference of *P* < 0.05 was considered statistically significant.

## Results

### Effect of tolvaptan (10 nM) on cAMP levels in MDCK cells: FRET‐based imaging

**Figure 1:**
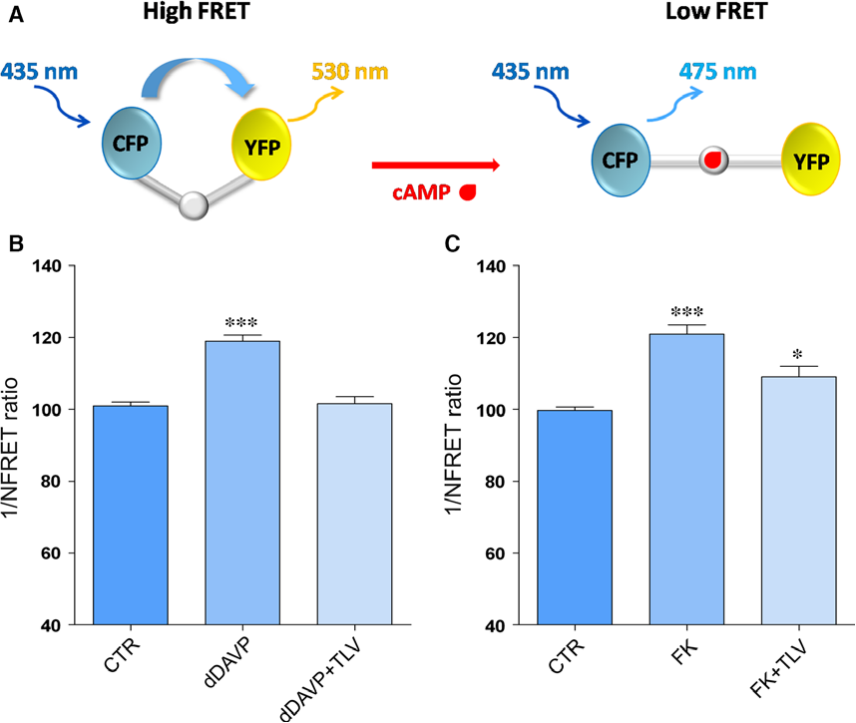
Evaluation of cAMP levels by fluorescence resonance energy transfer (FRET) analysis. (**A**) Schematic model showing a FRET probe containing cAMP‐binding sequence of Epac1 sandwiched between ECFP (donor) and EYFP (acceptor). Binding of cAMP to the Epac1 results in an intermolecular steric conformation causing an increase in the distance between the fluorescent donor and the acceptor, decreasing FRET process. (**B**) Histogram (means ± S.E.Ms; ***P < 0.0001) compares changes in normalized FRET reciprocal ratio (1/NFRET) with the cytosolic EPAC‐based cAMP FRET sensor (H96) under basal (CTR, *n* = 120 cells), dDAVP (*n* = 84 cells) and dDAVP with tolvaptan (dDAVP+TLV, *n* = 96 cells) treatments. (**C**) Histogram (means ± S.E.Ms; ***P < 0.0001 *versus* CTR; *P < 0.01 *versus* CTR) compares changes in normalized FRET reciprocal ratio (1/NFRET) with the membrane target EPAC‐based cAMP FRET sensor (H30mp) under basal (CTR, *n* = 30 cells), forskolin (FK, *n* = 37 cells) and forskolin with tolvaptan (FK+TLV, *n* = 30 cells) treatments.

Previous studies have shown that tolvaptan impairs the increase in cAMP levels upon vasopressin action 27. Here, the effect of tolvaptan on cAMP level was evaluated with FRET‐based EPAC biosensors as already described 28. Specifically, the FRET probe contains cAMP‐binding sequence of Epac1 sandwiched between ECFP (donor) and EYFP (acceptor). Binding of cAMP to the Epac1 causes an intermolecular steric conformation change, resulting in a relevant increase in the distance between the fluorescent donor and the acceptor, decreasing the FRET process (12, Fig. 1A). MDCK‐hAQP2 (clone I) cells, known to express endogenous functional V2R 16, were exposed to dDAVP or FK stimulation in the presence or in the absence of tolvaptan (10 nM). 10 nM tolvaptan was chosen as the appropriate minimal concentration displaying a clear inhibitory effect on dDAVP‐induced increase in cAMP levels in MDCK cells (not shown). In cells exposed to dDAVP, a clear increase in cAMP, depicted as an increase in the 1/NFRET ratio, was observed (Fig. 1B). As expected, tolvaptan treatment prevented dDAVP‐induced raise in cAMP levels (Fig. 1B). The data are in agreement with previous finding obtained in renal cell cultures, showing that tolvaptan causes a concentration‐dependent inhibition of vasopressin‐induced cAMP production 29. To further confirm the specificity of tolvaptan action as V2R antagonist, its effect on cAMP levels was evaluated in response to FK, a direct activator of the adenylate cyclase. In cells exposed to FK, an increase in cAMP, depicted as an increase in the 1/NFRET ratio, was observed (Fig. 1C). Unexpectedly, tolvaptan induced a slight although significant reduction in the FK‐induced increase in cAMP levels which, however, remained significantly higher compared with controls (Fig. 1C). It has to be underlined that, due to the highly diffusible nature of cAMP, the experiments performed with the generic adenylate cyclase activator FK were performed with the FRET biosensor H30 that is targeted to the plasma membrane and, therefore, sensitive to the submembrane cAMP microdomains 22.

### Tolvaptan prevents AQP2 translocation to the plasma membrane and the increase in pS256‐AQP2 in response to dDAVP in MDCK and in mouse kidney slices

**Figure 2:**
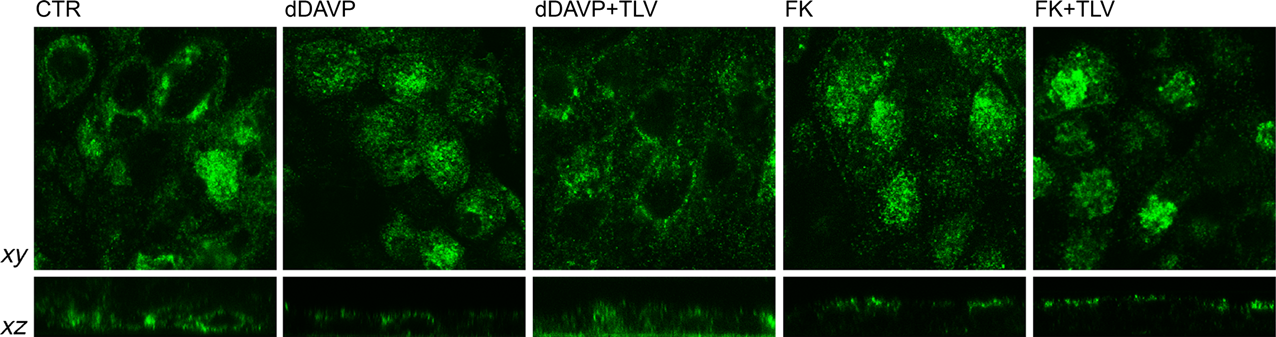
Effect of tolvaptan on AQP2 localization in MDCK‐hAQP2 cells. MDCK cells were grown on filters and were exposed to different treatments. AQP2 was localized by confocal microscopy. Stimulation with dDAVP caused AQP2 translocation to the apical plasma membrane. In contrast, tolvaptan prevented dDAVP‐induced AQP2 redistribution (dDAVP+TLV). Apparently tolvaptan did not prevent the re‐localization of the bulk of AQP2 to the apical membrane in response to FK.

The effect of tolvaptan on dDAVP‐induced AQP2 trafficking was analysed by confocal microscopy. Compared with controls (CTR) showing an intracellular AQP2 localization, dDAVP stimulation caused an increase in cell surface expression of AQP2 at the apical plasma membrane. Conversely, treatment with tolvaptan prevented AQP2 relocation to the plasma membrane associated with dDAVP stimulation (Fig. 2, dDAVP+TLV). FK stimulation had an apparent effect similar to dDAVP stimulation on AQP2 redistribution to the plasma membrane (Fig. 2, FK). Tolvaptan appeared to not significantly prevent the bulk of AQP2 redistribution to the apical plasma membrane (Fig. 2, FK+TLV).

**Figure 3:**
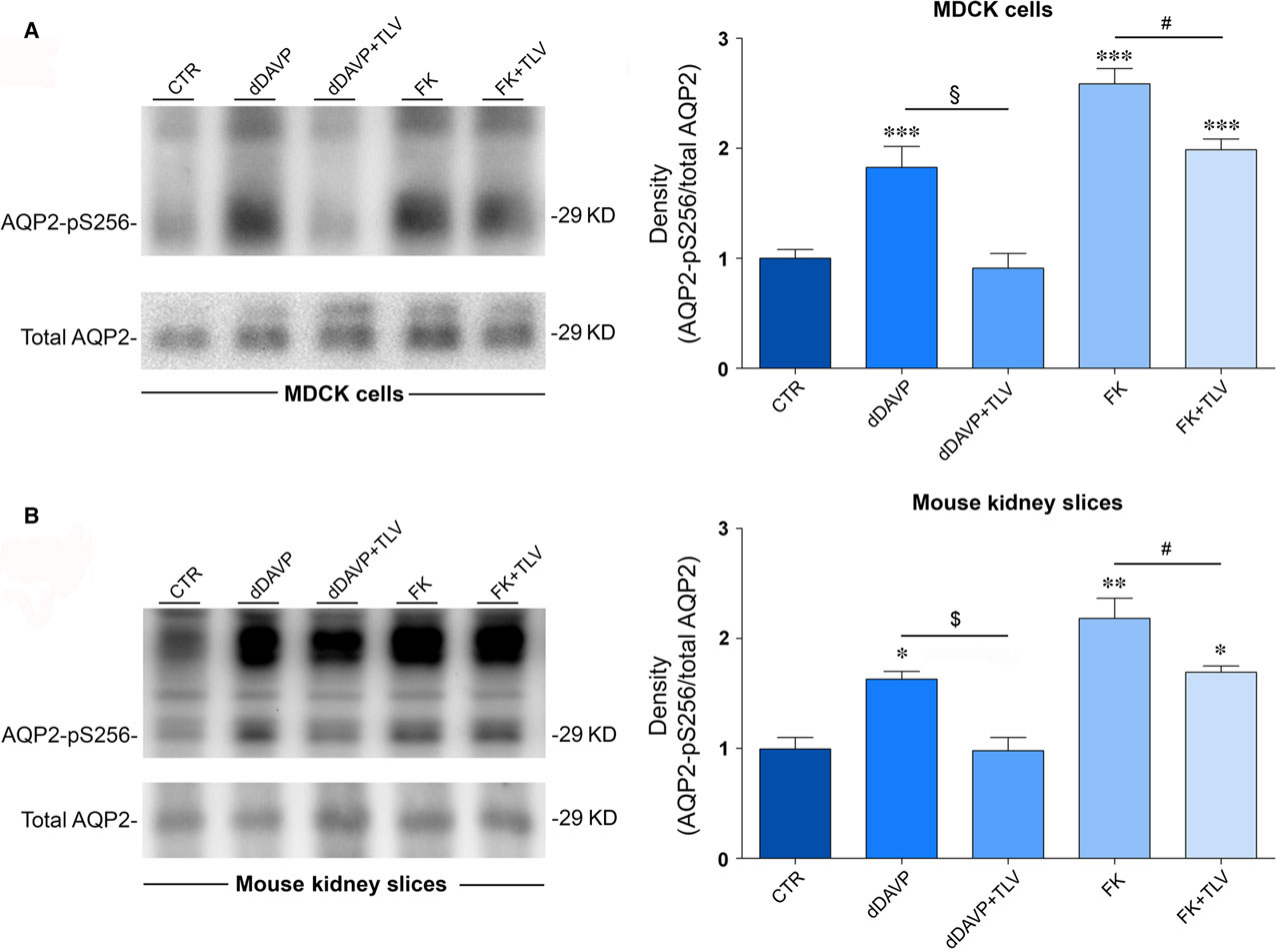
Effect of tolvaptan on AQP2 S256 phosphorylation in MDCK‐hAQP2 lysates and mouse kidney slices. (**A**) MDCK‐hAQP2 cells were treated as described under concise methods. Equal amount of proteins from cells (30 μg) were immunoblotted for total AQP2 and AQP2 phosphorylated at S256 (AQP2‐pS256). Signals were semiquantified by densitometry (right panel). Statistical analysis (means ± S.E.Ms; ***P < 0.0001 *versus* CTR; §*P* < 0.0001 dDAVP *versus* dDAVP+TLV; #*P* < 0.01 FK *versus* FK+TLV; *n* = 6) revealed that tolvaptan prevented the increase in AQP2‐pS256 compared with dDAVP and forskolin stimulation. (**B**) Mouse kidney slices were treated as described above. Equal amount of proteins from cells (15 μg) were immunoblotted for total AQP2 and AQP2 phosphorylated at S256 (AQP2‐pS256). Signals were semiquantified by densitometry (right panel). Statistical analysis (means ± S.E.Ms; *P < 0.01 *versus* CTR; **P < 0.001 *versus* CTR; $*P* < 0.01 dDAVP *versus* dDAVP+TLV; #*P* < 0.01 FK *versus* FK+TLV; *n* = 3) revealed that tolvaptan prevented the increase in AQP2‐pS256 compared with dDAVP and forskolin stimulation.

As AQP2 phosphorylation at ser256 is a prerequisite for its relocation to the plasma membrane, the effect of tolvaptan on pS256‐AQP2 was next evaluated in MDCK cells. With respect to control, dDAVP significantly increased pS256‐AQP2. Pre‐treatment with tolvaptan prevented the increase in pS256‐AQP2 consistent with its action as V2R antagonist (Fig. 3A). Tolvaptan alone had no apparent effect on basal levels of pS256‐AQP2 (data not shown). Consistent with the data of cAMP levels, tolvaptan partially reduced the increase in pS256‐AQP2 induced by FK in MDCK cells (Fig. 3A).

To test whether the inhibitory effect of tolvaptan on dDAVP‐induced pS256‐AQP2 also occurs in native kidney, *ex vivo* experiments were performed in fresh mouse kidney slices incubated with tolvaptan in the presence or in the absence of dDAVP. Compared with untreated tissue, pre‐incubation with tolvaptan prevented the dDAVP‐dependent increase in pS256‐AQP2, demonstrating that the drug is also effective in mouse kidney (Fig. 3B). Similar to what was observed in MDCK cells, tolvaptan partially reduced the increase in pS256‐AQP2 induced by FK in mouse kidney slices as well (Fig. 3B).

### Tolvaptan increases basal Ca_i_^+2^ and reduces the increase in Ca_i_^+2^ induced by dDAVP in MDCK

**Figure 4:**
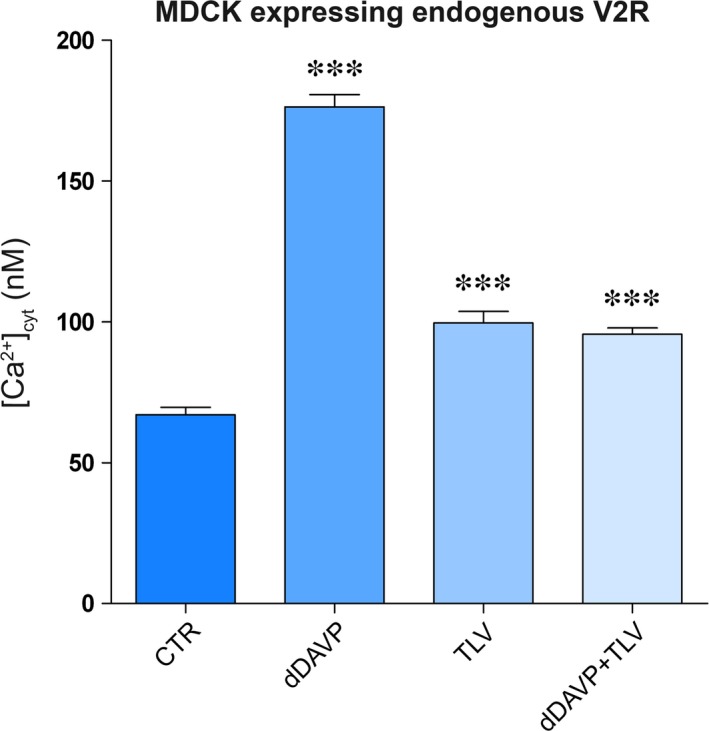
Evaluation of intracellular calcium content. MDCK‐hAQP2 cells were loaded with 7 μM fura‐2‐AM for 15 min. at 37°C in DMEM. Fluorescence measurements were taken with Metafluor software (Molecular Devices, MDS Analytical Technologies). Free cytosolic [Ca^2+^] was calculated according to Grynkiewicz formula as described in concise methods and measured in cells left under basal conditions (CTRL, *n* = 116 cells), or treated with dDAVP (*n* = 47 cells), TLV (*n* = 127 cells), or dDAVP in the presence of TLV (*n* = 44 cells). Data are expressed as means ± S.E.Ms (***P < 0.0001 *versus* CTR).

**Figure 5:**
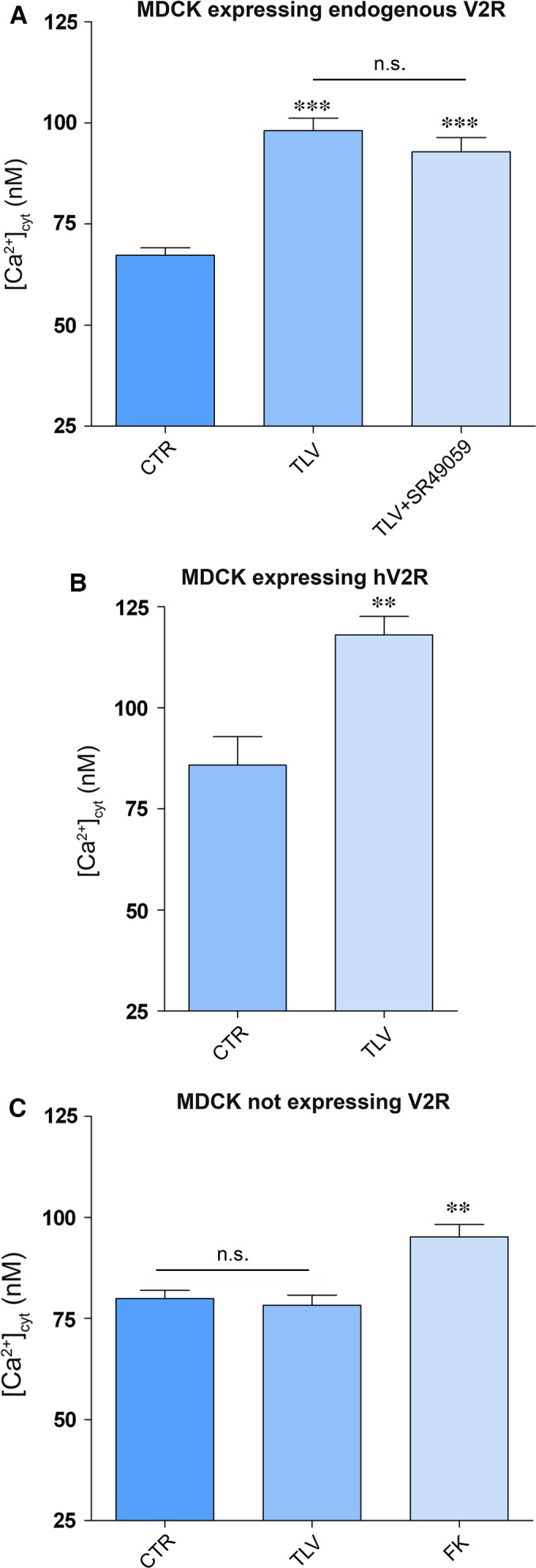
Effect of tolvaptan on free cytosolic calcium content in MDCK cells. (**A**) MDCKI cells, expressing V2R endogenously, were loaded with 7 μM fura‐2‐AM for 15 min. at 37°C in DMEM. Cells were left under basal conditions (*n* = 71 cells) or treated with tolvaptan (*n* = 97 cells) or tolvaptan in the presence of V1a receptor antagonist (SR49059) (*n* = 60 cells). Data are expressed as means ± S.E.Ms (***P < 0.0001 *versus* CTR). (**B**) MDCKI cells were transiently transfected with human V2 receptor, EGFP tagged. Free cytosolic [Ca^2+^] was calculated 48 hrs after transfection. Tolvaptan‐treated cells (*n* = 27 cells) showed a significant increase in calcium content with respect to untreated cells (*n* = 20 cells). Data (means ± S.E.Ms) were analysed by Student's *t* test with **P < 0.0003 considered to be statistically different. (**C**) MDCK cells clone II, not expressing V2R, were left untreated (*n* = 76) or stimulated with tolvaptan (*n* = 107) or with forskolin (*n* = 30) and subjected to intracellular calcium measurements. Data are expressed as means ± S.E.Ms (**P < 0.001 *versus* CTR).

Vasopressin triggers intracellular calcium mobilization from ryanodine‐sensitive calcium stores which is coupled to the apical exocytotic insertion of AQP2 30. Therefore, we next evaluated the effect of tolvaptan on dDAVP‐associated calcium signalling. Interestingly, calibration of intracellular calcium in MCDK cells revealed that tolvaptan *per se* caused a significant increase in intracellular calcium (Fig. 4; TLV 99.6 ± 4.1 nM; ctr 67.06 ± 2.6 nM, *P* < 0.0001). On the other hand, dDAVP action significantly increased the intracellular calcium concentration (176.4 ± 4.3 nM, *P* < 0.0001), while tolvaptan pre‐treatment significantly reduced this effect, although calcium levels did not return to baseline but reached levels similar to that found in cells treated with tolvaptan alone (Fig. 4). Tolvaptan has been proved to bind V2R receptor 29 times more efficiently than the V1aR, a receptor whose activation promotes intracellular calcium increase 27. To exclude that the increase in intracellular calcium associated with tolvaptan treatment was due to a possible unknown agonistic effect on V1aR, MDCK cells were pre‐treated with the V1aR inhibitor SR49059. Under these experimental conditions, tolvaptan still elicited a significant increase in intracellular calcium which was not significantly different from the condition of TLV alone, indicating that this effect is specifically associated with tolvaptan action on V2R receptor (Fig. 5A).

Tolvaptan has been proved to be effective as a specific agonist of the rat and human V2R 31, 32. As MDCK cells are of dog origin, we evaluated whether the increase in intracellular calcium induced by tolvaptan is also observed in cells expressing human V2R. MDCK cells clone II (not expressing V2R) were transiently transfected with human V2R (h‐V2R), and the effect of tolvaptan on intracellular calcium was evaluated. Tolvaptan treatment caused a significant increase in intracellular calcium (TLV 118.1 ± 4.6 nM; ctr 85.87 ± 4.7, *P* < 0.0003), indicating that this effect is not an artefact related to the endogenous V2R expressed in MDCK of dog origin (Fig. 5B). Finally, to further confirm that the effect of tolvaptan in elevating intracellular calcium is strictly dependent on its interaction with the V2R, its action was also tested in MDCK cells not expressing the V2R (clone II). No alteration in intracellular calcium was measured in this setting, clearly indicating that the cellular effect of tolvaptan in elevating intracellular calcium requires a functional V2 receptor (Fig. 5C).

### Tolvaptan prevents the increase in pS256‐AQP2 in response to dDAVP in kidney slices from V1aR −/− mice

**Figure 6:**
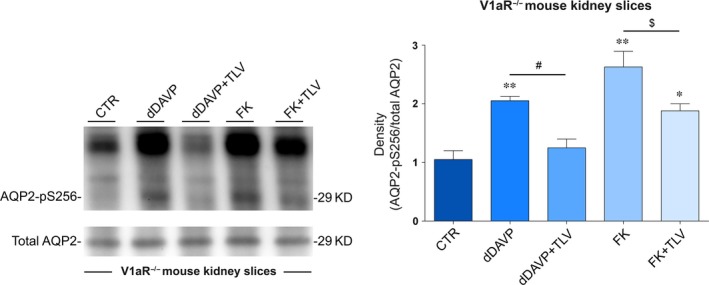
S256 phosphorylation of AQP2 in V1a receptor knockout mice. Fresh kidney slices were left untreated or stimulated as described in concise methods. Equal amount of proteins (15 μg) were immunoblotted with antibodies specific for total AQP2 or for AQP2‐pS256. Densitometric analysis (right panel) and statistical studies (means ± S.E.Ms) revealed that tolvaptan significantly prevented the increase in AQP2‐pS256 elicited by dDAVP (**P < 0.001 *versus* CTR; *P < 0.01 *versus* CTR; #*P* < 0.01 dDAVP *versus* dDAVP+TLV; $*P* < 0.01 FK *versus* FK+TLV; *n* = 3).

As the cellular effect of tolvaptan on intracellular calcium may have a relevant clinical implication, we further investigated the possible contribution of the V1aR‐Ca2 + pathway in response to tolvaptan in mice kidney lacking the V1aR (V1aR −/− mice 33). Specifically, the effect of tolvaptan on pS256‐AQP2 levels in response to dDAVP was evaluated in V1aR −/− mice inner medulla kidney slices. Results confirm that, compared with untreated tissue, pre‐incubation with tolvaptan prevented dDAVP‐dependent increase in pS256‐AQP2, demonstrating that the antagonist had the same effect in V1aR −/− mice as in wild‐type mice, thus excluding any role of the V1aR expressed in the kidney in the tolvaptan response (Fig. 6). Moreover, tolvaptan, similar to what is observed in wild‐type mouse kidney slides, slightly but significantly reduced the increase in pS256‐AQP2 induced by FK in V1aR −/− mice (Fig. 6).

### Tolvaptan induces release of calcium from ER

**Figure 7:**
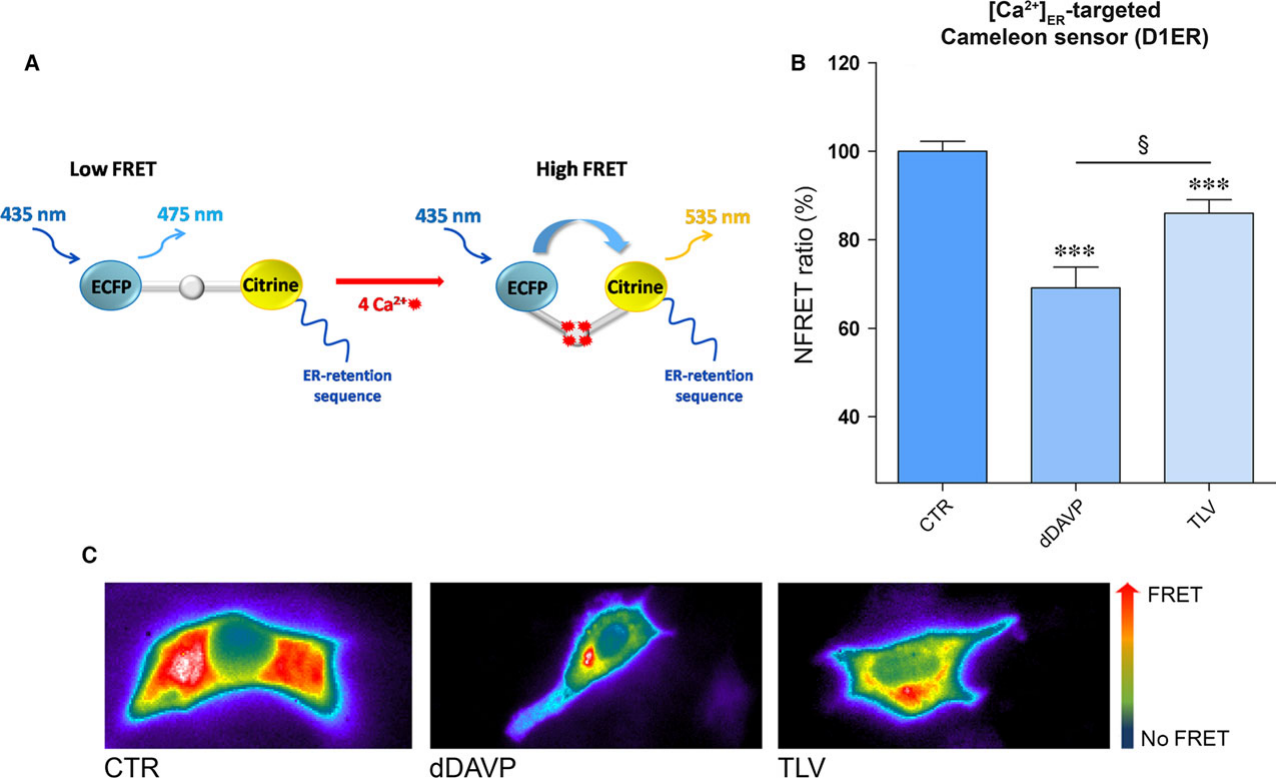
Evaluation of [Ca^2+^]_ER_ with ER‐targeted Cameleon (D1ER) probe. (**A**) Model showing the Cameleon (D1ER) probe. Binding of calcium to calmodulin sequence results in an intramolecular rearrangement of the probe leading to an increase in NFRET signal. (**B**) Histogram (means ± S.E.Ms; ***P < 0.0001 *versus* CTR; §*P* < 0.0001 dDAVP *versus* TLV) compares changes in normalized FRET (NFRET) ratio between control (*n* = 52 cells), dDAVP (*n* = 50 cells)‐ and tolvaptan (*n* = 49 cells)‐treated cells. (**C**) Representative transfected cells with the ER‐targeted cameleon showing the FRET signal (ratio of 535/480 nm) which is depicted in false colors.

We next asked the question of whether tolvaptan increases intracellular calcium by promoting calcium release from intracellular stores. Dynamics of calcium in the ER were tested with a genetically encoded Cameleon sensor, the ER‐targeted Cameleon (D1ER) probe containing a ER retention motif, which detects [Ca^2+^]_ER_ directly (Fig. 7A) 23, 34. FRET studies showed that exposure of cells to dDAVP caused a release of calcium from the ER depicted by a significant reduction in the FRET signal. This is in agreement with previous data demonstrating that vasopressin signalling causes calcium release from ER 30. Interestingly, tolvaptan induced a significant reduction in calcium accumulation in the ER (86 ± 3.04% *versus* ctr 100 ± 2.26%, **P < 0.0001), suggesting that tolvaptan causes *per se* a release of calcium from the ER (Fig. 7B, TLV).

### Tolvaptan treatment is associated with a significant decrease in protein phosphatases 1 activity

**Figure 8:**
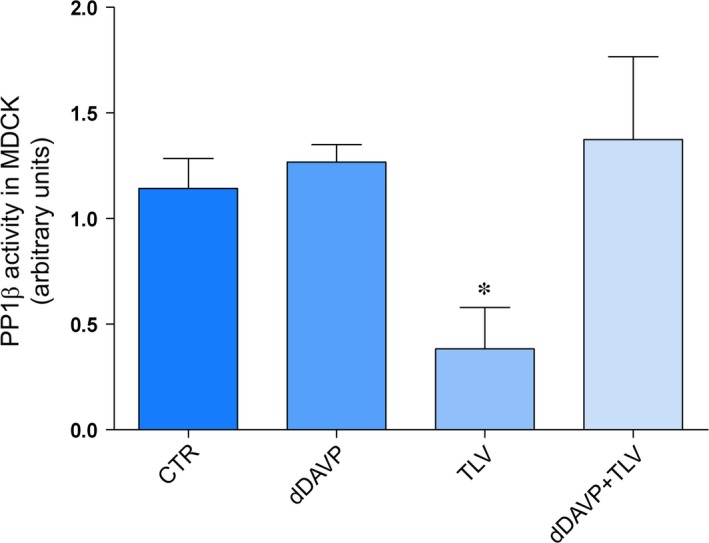
Effect of tolvaptan on PP1β activity. Cells were left under basal conditions, or treated as indicated above. Protein phosphatase activity was evaluated with an immunoprecipitation assay kit as described in concise methods. Data are expressed as means ± S.E.Ms (*P < 0.01 *versus* CTR; *n* = 3).

The possible molecular basis of the tolvaptan‐induced calcium release from the ER was next evaluated. Altered calcium handling has been associated with dysregulated protein phosphatase 1 (PP1). PP1 regulates different subcellular targets including the ryanodine receptor (RyR). RyR2 is phosphorylated by PKA, and the phosphorylation of RyR2 promotes calcium release from the ER, while PP1 dephosphorylates RyR2 35. Therefore, the activity of PP1 was investigated in MDCK cells in the presence and in the absence of tolvaptan. Quite interestingly, compared with control conditions, we found that tolvaptan caused *per se* a drastic significant decrease in PP1 activity (Fig. 8). The tolvaptan effect on PP1 was specific as no effect was observed on PP2A, a closely homologous phosphatase (data not shown).

### Tolvaptan treatment prevents the increase in osmotic water permeability in response to dDAVP and FK

**Figure 9:**
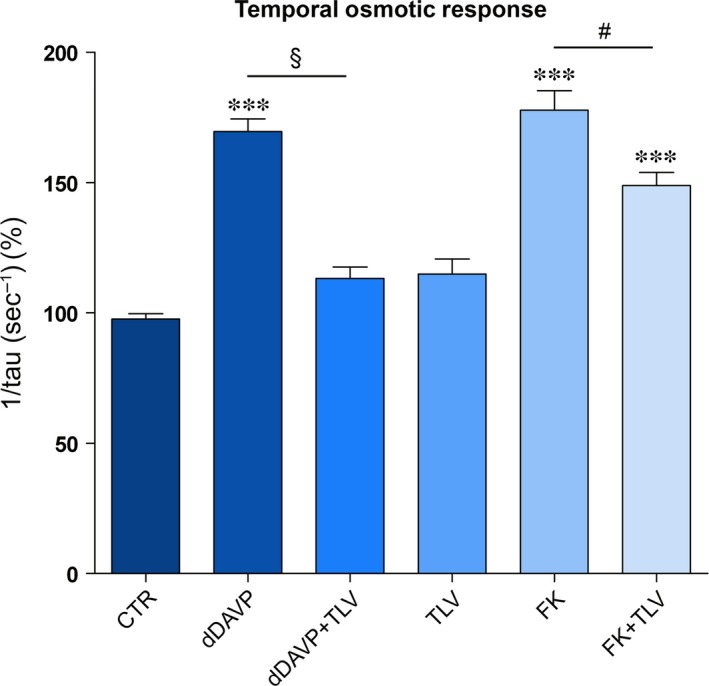
Time constant of cell swelling under hypertonic stimulus. Cells were treated as described in concise methods. The time course of fluorescence changes in calcein‐loaded cells indicates that tolvaptan impaired the dDAVP and forskolin induced cell swelling ability (means ± S.E.Ms). ***P < 0.001 *versus* CTR (*n* = 113 cells); §*P* < 0.0001 dDAVP (*n* = 72 cells) *versus* dDAVP+TLV (*n* = 55 cells); #*P* < 0.0001 FK (*n* = 91 cells) *versus* FK+TLV (*n* = 115 cells).

AQP2 phosphorylation at ser256 is required for its insertion into the plasma membrane leading to an increase in water permeability. The effect of tolvaptan on the time course of the osmotic water permeability in response to dDAVP was next investigated in MDCK cells, by video imaging experiments. Cells were grown on Ø40‐mm glass coverslips and loaded with membrane‐permeable calcein‐AM. Fluorescence measurements were taken in the presence of either iso‐ or hyperosmotic solutions. dDAVP treatment induced a significantly higher temporal osmotic response (reported as 1/τ) as compared to untreated cells (169.6 ± 4.8 *versus* ctr = 100 ± 2, arbitrary units, *P* < 0.0001, Fig. 9). Tolvaptan alone did not affect the basal osmotic water permeability (Fig. 9).

Conversely, as expected for a V2R antagonist, tolvaptan prevented the increase in the osmotic water permeability in response to dDAVP, indicating that tolvaptan impairs AQP2 insertion to the plasma membrane (Fig. 9). In line with data obtained for cAMP and p256‐AQP2 levels, tolvaptan significantly reduced (about 16%, *P* < 0.0001) the increase in the osmotic response elicited by FK stimulation consistent with the hypothesis that tolvaptan has a post‐receptor effect. This effect, however, may contribute to the overall reduction in the osmotic vasopressin response and, therefore, to the aquaretic effect (Fig. 9). The osmotic response was also tested in the presence of vinpocetine, a selective inhibitor of the calcium‐activatable phosphodiesterase 1 (PDE1) 36. Treatment with vinpocetine alone increased the osmotic response due to the increase in cAMP and, in turn, of pS256‐AQP2. Tolvaptan did not prevent the increase in the osmotic response elicited by vinpocetine (data not shown). This result indicates that, in contrast to data obtained with FK, tolvaptan does not manifest any antagonist effect if the raise in cAMP is obtained by stimulating a post‐receptor/AC target.

### Tolvaptan reduces aquaporin 2 excretion in patients with SIAD and normalizes serum sodium levels

Tolvaptan promotes the excretion of electrolyte‐free water, known as aquaresis, and is authorized for the treatment of hyponatraemia due to the syndrome of inappropriate antidiuretic hormone secretion (SIAD), a common cause of hyponatraemia. However, no data are so far available in humans to demonstrate that the aquaretic effect of tolvaptan is associated with reduction in AQP2 excretion in SIAD, reflecting an impairment of AQP2 trafficking *in vivo*. AQP2 excretion was tested in two patients whose clinical and laboratory evaluations were consistent with the presence of SIAD. Before tolvaptan treatment (15 mg/day) patient 1 (59 years old, male), urinary osmolality was 435 mOsm, urine sodium was 171 mM and plasma sodium was 132 mM. It has to be pointed out that these values were measured while the patient 1 was under hypertonic treatment to maintain serum sodium to 132 mM, otherwise it, most likely, would have been much lower.

Patient 2 (81 years old, female), before tolvaptan treatment (15 mg/day), had a urinary osmolality of 317 mOsm, urine sodium was 67 mM and plasma sodium was 115 mM (patient 2 was not under hypertonic saline).

**Figure 10:**
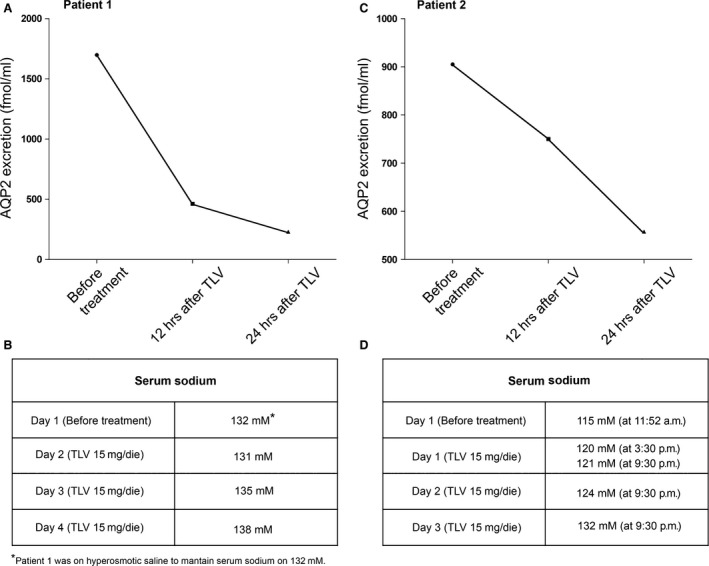
Effect of tolvaptan on urinary excretion of AQP2. (**A**) Evaluation of urinary AQP2 in patient 1 affected by SIAD after 12‐h and 24‐h tolvaptan administration. (**B**) Effect of tolvaptan treatment on natraemia of patient 1. (**C**) Evaluation of urinary AQP2 in patient 2 affected by SIAD after 12‐h and 24‐h tolvaptan administration. (**D**) Effect of tolvaptan treatment on natraemia of patient 2.

On day 1 before treatment, both patients had a relatively high AQP2 excretion compared with the average AQP2 excretion found in healthy individuals. In patient 1, a drastic reduction in AQP2 excretion was seen already after 12 hrs (73%, from 1698 to 463 fmol/l) with further reduction after 24 hrs, reaching values corresponding to about 14% of the initial value (231 fmol/ml; Fig. 10A and B). The strong reduction in AQP2 excretion was consistent with the aquaretic effect of tolvaptan leading to reduced water retention with a parallel increase in serum sodium levels which reached the value of 138 mM at day 4.

In patient 2, AQP2 excretion decreased by 17% (from 905 to 750 fmol/l) after 12 hrs with further strong reduction after 24 hrs reaching the values of 558 fmol/ml (Fig. 10C and D). Again the reduction in AQP2 excretion was paralleled by a progressive increase in serum sodium which reached the value of 132 mM at day 3 and remained stable at 135 mM after 3 months of treatment (Fig. 10C and D).

To our knowledge, these are the first evidence of the tolvaptan effect on AQP2 excretion in SIAD patients.

## Discussion

The major findings of this study can be summarized as follows: (*i*) We provide *in vitro* evidence that tolvaptan *per se* causes calcium mobilization from the ER resulting in a significant increase in basal intracellular calcium; (*ii*) we demonstrate that tolvaptan counteracts vasopressin‐induced AQP2 relocation to plasma membrane and inhibits osmotic water transport in collecting duct principal cells expressing endogenous V2R receptor; (*iii*) we report that tolvaptan reduces AQP2 excretion in two SIAD patients paralleled by normalization of plasma sodium concentration.

Although tolvaptan has been shown to induce aquaresis, resulting in an amelioration of hyponatraemia in patients with cirrhosis, congestive heart failure and SIAD 37, 38, 39, 40, to date no direct *in vitro* evidence has been provided that tolvaptan impairs AQP2 trafficking and insertion to the plasma membrane in collecting duct principal cells in response to vasopressin thus blunting AQP2‐mediated osmotic water permeability. In this respect, a global analysis of the *in vitro* effect of tolvaptan on rat collecting duct suspension showed that tolvaptan inhibits vasopressin effect on AQP2 phosphorylation at ser256, ser264 and ser269 while inhibiting ser261 10. Moreover, quantitative phosphoproteomics of rat inner medulla collecting duct principal cells revealed that several phosphopeptides including AQP2 were significantly changed by another V2R‐specific antagonist, satavaptan. Specifically, AQP2 phosphorylation at residues S256, S264 and S269 was significantly reduced, while phosphorylation at residue S261 increased in cells exposed to dDAVP after pre‐incubation with satavaptan 41. Although these data provide a general indication that a V2R‐specific antagonist affects AQP2 phosphorylation counteracting the physiological vasopressin effects on these post‐translational modifications, the final evidence for an impairment of AQP2 relocation to the plasma membrane with consequent inhibition of osmotic water transport is lacking for tolvaptan as well as for satavaptan. Here, we demonstrate that tolvaptan prevents dDAVP‐induced ser256‐AQP2 phosphorylation both in MDCK cells expressing endogenous V2R and in native mouse inner medulla kidney slices (Fig. 3) and this effect is associated with an impairment of AQP2 relocation to the plasma membrane and inhibition of the osmotic water permeability (Fig. 9). Of note, we show that tolvaptan prevents the increase in pS256‐AQP2 in response to dDAVP also in kidney slices from V1aR −/− mice, thus excluding the involvement of the V1aR expressed in the kidney in the tolvaptan response.

These results clarify the precise cellular mechanism of the tolvaptan action and represent the definitive proof that the water channel AQP2 is the functional target of this V2R antagonist.

In line with these findings, recent data indicate that AQP2 excretion can be considered a novel non‐invasive marker to predict the response to tolvaptan in patients with congestive and decompensated heart failure 42, 43 and has recently been found to correlate with the pharmacological effect of tolvaptan in cirrhotic patients with ascites 44. Three per cent of the renal AQP2 is in fact excreted in the urine and is proportional to the AQP2 reaching the apical plasma membrane in response to vasopressin 45. The V2 receptor antagonist VPA‐985 was also found to decrease urinary AQP2 in patients with chronic heart failure 46. Here, we show that tolvaptan is efficacious in raising serum sodium levels in two SIAD patients and this effect was paralleled by a rapid and dramatic reduction in AQP2 excretion (to about 80% and 40% of the value before the treatment in patients 1 and 2, respectively, after 24 hrs, Fig. 10). These data suggest that AQP2 can be a good and reliable marker for predicting response to tolvaptan in patients with SIAD.

### Unexpected cellular tolvaptan effects

Besides the demonstration that tolvaptan, indeed, prevents vasopressin‐induced AQP2 trafficking both *in vitro* and *in vivo*, a novel major observation of this study that may have a clinical relevance is that tolvaptan increases basal intracellular calcium in MDCK (Fig. 4). This effect was strictly dependent on the expression of the V2R, indicating that it requires tolvaptan–V2R interaction. The possible agonistic effect of tolvaptan *via* V1aR (coupled to calcium signalling) in MDCK cells was excluded as pre‐treatment with the V1aR inhibitor SR49059 did not prevent the effect of tolvaptan in raising intracellular calcium. In addition, this effect was also observed in MDCK cells transfected with the human V2R, thus excluding a possible artefact related to the endogenous V2R expressed in MDCK of dog origin. FRET studies showed that this increase in intracellular calcium was paralleled by a significant reduction in calcium accumulation in the ER, indicating that tolvaptan *per se* induces release of calcium from the ER (Fig. 7).

Calcium is released from the ER through the RyR2 which are regulated by their phosphorylation (promoting calcium release) and dephosphorylation by PP1 35. We show here that tolvaptan strongly decreases PP1 activity, an effect that was not observed under dDAVP treatment (Fig. 8), indicating that it is a specific effect associated with tolvaptan treatment. We may speculate that decreased PP1‐mediated dephosphorylation of RyR2 leads to RyR2 hyperactivity resulting in a release of calcium from the ER. Interestingly, local PP1 regulation of RyR2 resulting in enhanced phosphorylation of the receptor has been proposed to promote atrial fibrillation susceptibility in mice 35. Although in this work we do not address the mechanism of PP1 dysregulation under tolvaptan action, this finding may be of clinical interest.

The cellular effect of tolvaptan on calcium release from the ER might explain the surprising partial inhibitory effect of the V2R antagonist observed under FK action on cAMP levels (Fig. 1), AQP2‐ser256 (in both MCDK and rat kidney, Fig. 3) and on osmotic water permeability (Fig. 9). One possible explanation is that tolvaptan‐induced calcium release can partially contribute to the inhibitory action of tolvaptan on AQP2 trafficking hindering the calcium‐inhibitable AC6 expressed in collecting duct epithelial cells 47, 48, 49, 50, resulting in inhibition of the cAMP/PKA/AQP2 phosphorylation pathway. It is unlikely that tolvaptan causes cAMP reduction through activation of the calcium‐dependent PDE1 as we report here that tolvaptan did not prevent the increase in the osmotic response elicited by vinpocetine, a selective inhibitor of the PDE1 36.

Taken together, the data reported in this study represent the first demonstration of the central role of AQP2 blockade in the aquaretic effect of tolvaptan and underscore a novel tolvaptan effect on intracellular calcium. This novel observation might be important for the treatment of ADPKD, a disease for which tolvaptan, used at an early stage, has been shown to slow the progression of cysts formation and the decline of kidney function presumably by downregulating cAMP signalling 4. In line with these findings, genetic elimination of circulating vasopressin has been reported to inhibit the development of renal cysts in rat models 51. As the recent literature indicates that increased levels of cAMP in ADPKD are linked to the reduced intracellular calcium levels 52, the present observation that tolvaptan, besides reducing cAMP levels, increases intracellular calcium represents a medical advance of knowledge which might be relevant in the treatment of ADPKD.

## Conflict of interest

The authors confirm that there are no conflicts of interest.
